# Supplementary material for: Affinity Resins for the Isolation of Immunoglobulins G Obtained Using Biocatalytic Technology
Source: Int J Mol Sci. 2024 Jan 23;25(3):1367. doi: 10.3390/ijms25031367 (PMC10855859; doi:10.3390/ijms25031367)
Supplement: Supplementary file 1 [file ijms-25-01367-s001.zip › ijms-2795783-supplementary.pdf]

Supplementary Material

Affinity resins for isolation of immunoglobulins G obtained via biocatalytic technology

Mikhail N. Tereshin [1,2], Tatjana D. Melikhova [1], Barbara Z. Eletskaya [1], Olga B. Ksenofontova [1], Pavel V. Pantyushenko [1], Maria Ya. Berzina [1,*], Igor Ivanov [2], Igor V. Myagkikh [1] and Vasilij N. Stepanenko [1,2]

[1] M.M. Shemyakin and Yu.A. Ovchinnikov Institute of Bioorganic Chemistry, Russian Academy of Sciences (IBCh RAS), Miklukho-Maklaya street, 16/10, 117437, Moscow, Russia; misha060596@gmail.com (M. N. T.); tdm-63@yandex.ru (T. D. M); fraubarusya@gmail.com (B. Z. E.); labpeptos@gmail.com (O. B. K); pantyushenko.pavel@gmail.com (P. V. P.); berzina_maria@mail.ru (M. Ya. B.); myagkikh@ibch.ru (I. V. M.); svn@ibch.ru (V. N. S.)

[2] Lomonosov Institute of Fine Chemical Technologies, MIREA - Russian Technological University, Vernadskogo pr. 86, 119571 Moscow, Russia; igor_ivanov@gmx.de

Table of Content

^{1}H spectrum of compound Boc-Gly-Gly-Gly-Gly-OH (9)	S1
^{1}H spectrum of compound Boc-Gly-Gly-EDA-Gly-Gly-Boc (12)	S2

SI-1 Boc-Gly$_4$-OH

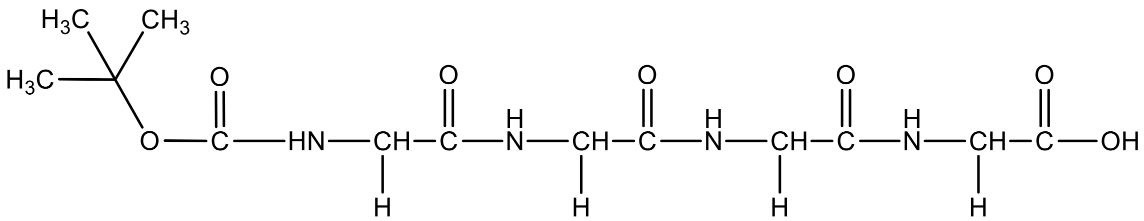

Chemical Formula: $C_{13}H_{22}N_4O_7$
Molecular Weight: 346,34

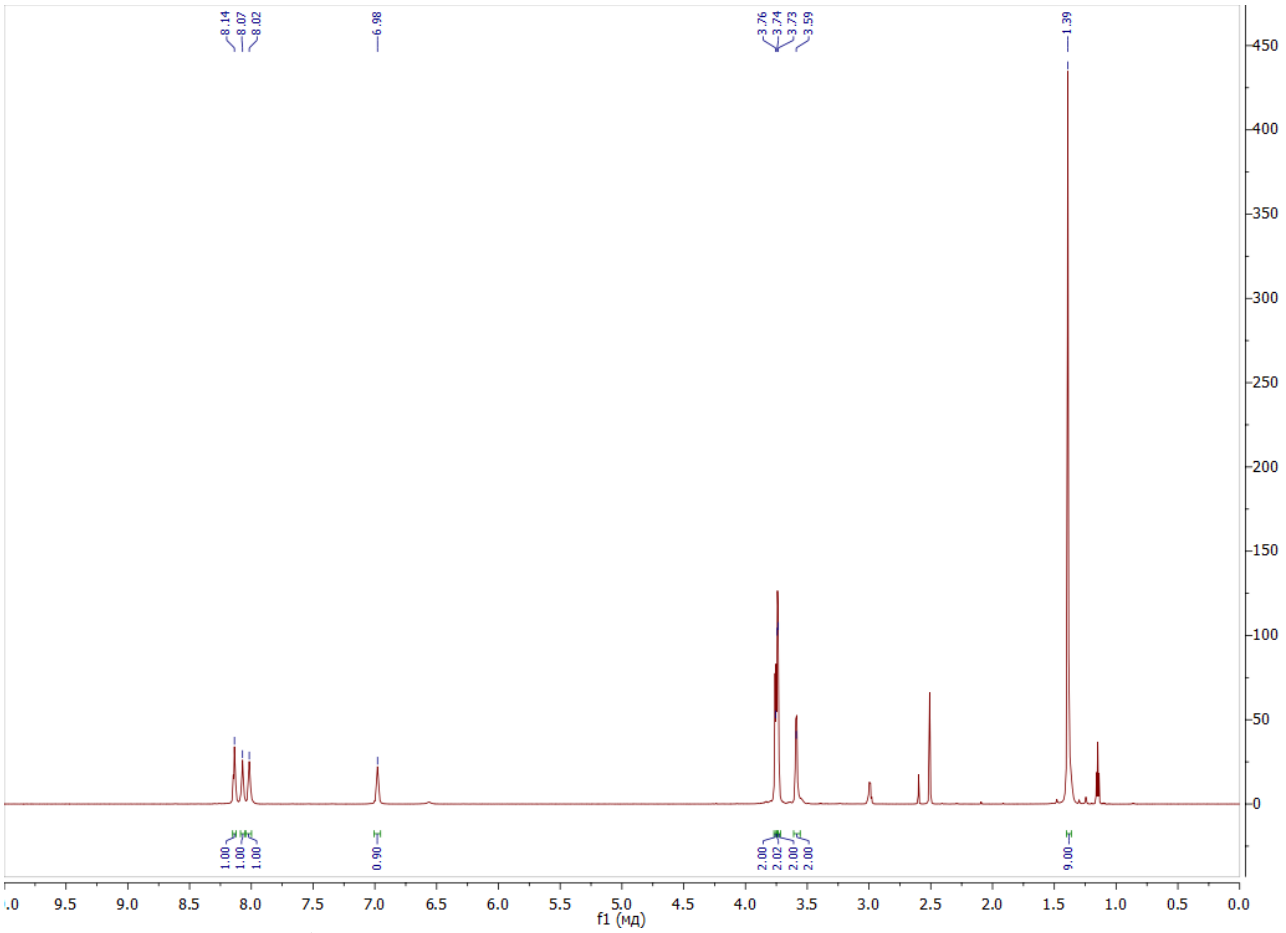

Figure S1. The ^{1}H NMR spectrum of Boc-Gly$_4$-OH

SI-2 Boc-Gly-Gly-EDA-Gly-Gly-Boc

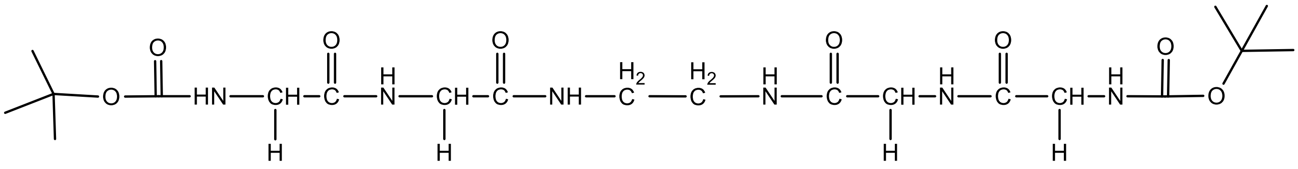

Chemical Formula: $C_{20}H_{36}N_6O_8$
Exact Mass: 488,26

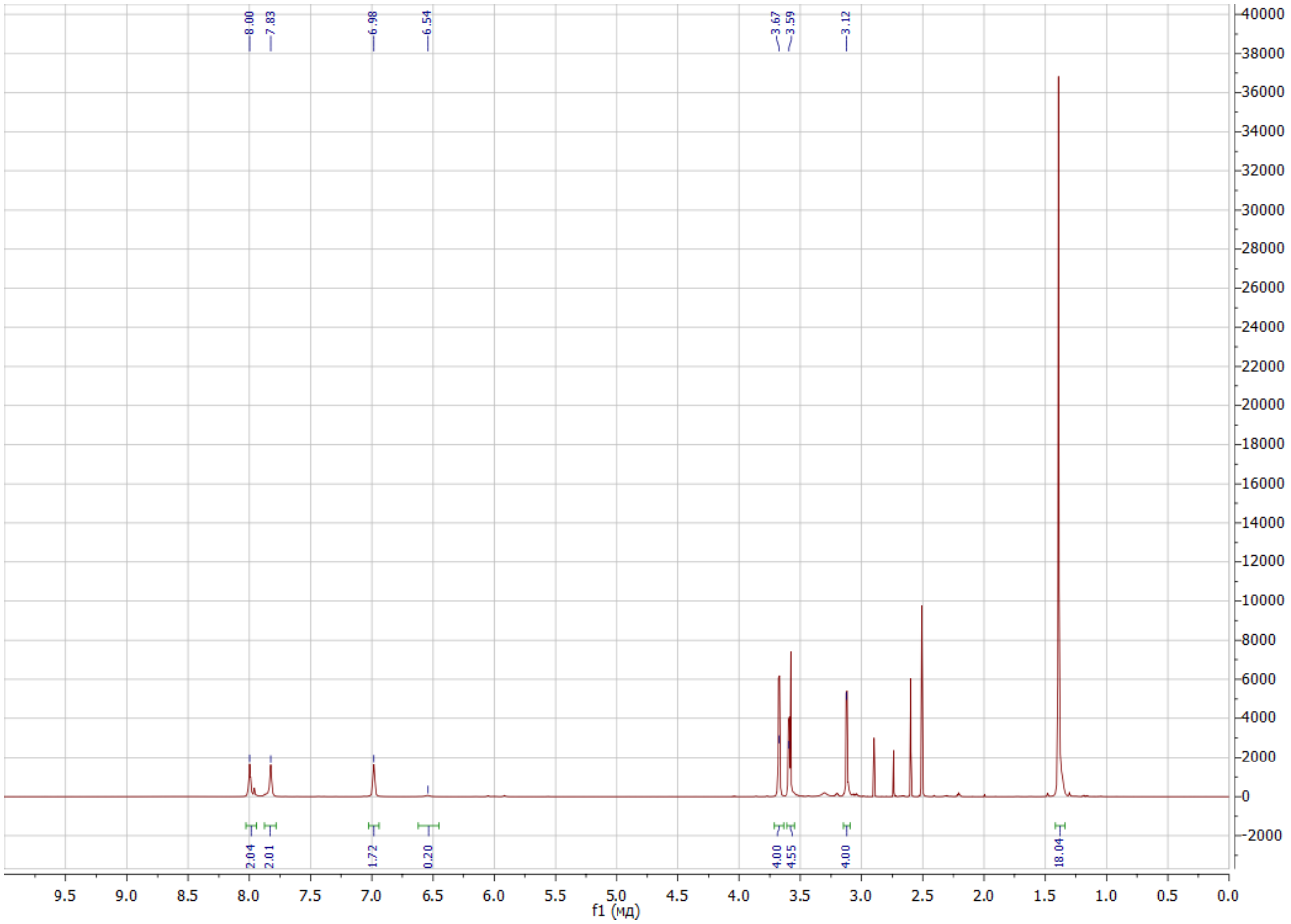

Figure S2. The ^{1}H NMR spectrum of Boc-Gly-Gly-EDA-Gly-Gly-Boc
